# Protein-Related Circular RNAs in Human Pathologies

**DOI:** 10.3390/cells9081841

**Published:** 2020-08-06

**Authors:** Olga Wawrzyniak, Żaneta Zarębska, Konrad Kuczyński, Anna Gotz-Więckowska, Katarzyna Rolle

**Affiliations:** 1 Department of Ophthalmology, Poznan University of Medical Sciences, 60569 Poznan, Poland; 55220@student.ump.edu.pl (O.W.); a.gotzwieckowska@gmail.com (A.G.-W.); 2 Department of Molecular Neurooncology, Institute of Bioorganic Chemistry Polish Academy of Sciences, 61704 Poznan, Poland; zzarebska@ibch.poznan.pl (Ż.Z.); kuczynski@ibch.poznan.pl (K.K.); 3 NanoBioMedical Centre, Adam Mickiewicz University, 61614 Poznan, Poland

**Keywords:** circRNAs, cancer, human disorders, translation, backsplicing

## Abstract

Circular RNAs (circRNAs) are a distinct family of RNAs derived from alternative splicing which play a crucial role in regulating gene expression by acting as microRNA (miRNA) and RNA binding protein (RBP) sponges. However, recent studies have also reported the multifunctional potential of these particles. Under different conditions, circRNAs not only regulate protein synthesis, destination, and degradation but can serve as protein scaffolds or recruiters and are also able to produce short peptides with active biological functions. circRNAs are under ongoing investigation because of their close association with the development of diseases. Some circRNAs are reportedly expressed in a tissue- and development stage-specific manner. Furthermore, due to other features of circRNAs, including their stability, conservation, and high abundance in bodily fluids, they are believed to be potential biomarkers for various diseases, including cancers. In this review, we focus on providing a summary of the current knowledge on circRNA–protein interactions. We present the properties and functions of circRNAs, the possible mechanisms of their translation abilities, and the emerging functions of circRNA-derived peptides in human pathologies.

## 1. Introduction

circRNAs comprise a novel class of regulatory RNAs that are abundant and highly conserved in mammals [1,2]. Circular forms of RNA were originally identified in the mid-1970s as plant viroids—uncoated, infectious, and covalently closed RNAs. However, for a long time, no attention was paid to the potential functions that these RNAs may perform [3,4]. Interestingly, certain human circular transcripts tend to be expressed at higher levels than their linear counterparts, a fact that prompted a cascade of research projects that eventually led to a breakthrough in circRNA research [5,6].

The development of advanced sequencing techniques, especially RNA-seq, has allowed thousands of circRNAs to be identified in various organisms. In previous studies, circRNAs, like other RNAs without poly(A) tails, were depleted during poly(A) selection as a routine step during RNA-seq library preparation. The development of RNA purification methods, such as ribosomal RNA and poly(A) depletion, allowed researchers to obtain RNA-seq libraries enriched in the circRNA fraction [7]. The diversity of genomic locations that may give rise to circular transcripts has an impact on their specific expression patterns. Previously, it was postulated that circRNAs are generated based on the tissue, cell type, and developmental stage, which could be highly connected with the functions these RNAs perform in particular environments [8,9,10]. Research has shown the aberrant expression patterns of circular transcript groups under disease conditions, indicating a significant role of circRNAs in basic cellular events and pathogeneses that allows circRNAs to be considered as a putative target for disease diagnosis and treatment [11,12,13].

Investigating the interactions between circRNAs and proteins is a fascinating and exciting quest. This publication presents the current knowledge about circRNAs’ abilities to act in multiple layers of protein formation, maturation, and activity. Whether circRNAs serve as a template for production or as decoy particles, how they regulate essential biological processes, and what their role is under pathological conditions remain open questions.

## 2. Biogenesis of circRNAs

The biogenesis of circRNAs is connected to the canonical splicing of pre-mRNAs, leading to the specific interplay between both events. Canonical splicing and circRNA biogenesis are precisely controlled by the existence of many cis-acting elements and trans-acting factors to maintain the physiological balance within the cell [14].

### 2.1. Cis-Acting Elements

**Figure 1:**
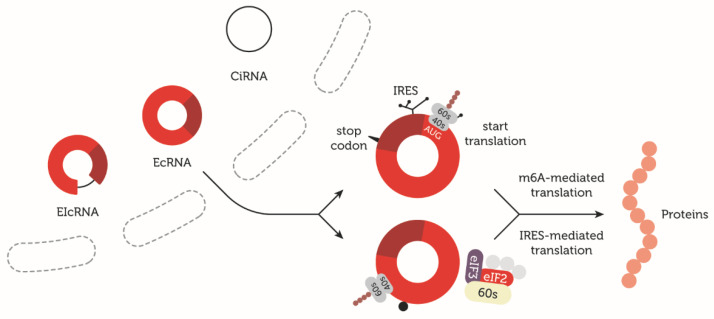
circRNAs can be classified based on their origin as exonic (EcRNA), intronic (CiRNA), and exon-intron (EIcRNA) transcripts. EcRNAs are mostly exported from the nucleus to the cytoplasm, and some can serve as a template for protein synthesis. In eukaryotic cells, two potential mechanisms of the cap-independent translation of circRNAs are proposed: the Internal Ribosome Entry Site (IRES) and N^6^-methyladenosines (m^6^A). In the cap-independent mechanism of translation initiation, a non-canonical eIF4G protein needs to recognize the IRES to initiate eIF4 complex assembly in order to initiate the translation. Translation initiation with the presence of m^6^A requires YTH domain-containing family protein 3 (YTHDF3), an m^6^A reader protein that can directly interact with eIF4G2, which recognizes IRESs and initiates the eIF4 complex assembly.

Repetitive and nonrepetitive flanking intronic complementary sequences are the most common motifs representing cis-acting elements, where the pairing of complementary nucleotides leads to circularization. While circRNAs generally comprise 1–5 exons flanked by intronic sequences, the existence of exonic, intronic, exon-intronic, and intergenic sequences has been demonstrated, as shown in Figure 1 [15,16]. Two basic models of circRNA biogenesis are presented in the literature: direct backsplicing and the lariat model (or exon skipping model), where the canonical splicing of pre-mRNA occurs first. In the backsplicing model, the circRNA is generated first, but the process still requires both canonical spliceosomal machinery and canonical splice signals [14]. The lariat model is considered a more comprehensive mechanism of circRNA biogenesis due to the series of events leading to the generation of properly closed circular molecules. The major difference between both processes is the necessity for lariat intermediate processing after the canonical splicing of pre-mRNAs. The result is covalent binding of the 5′ site of an upstream exon with the 3′ of the same exon or a downstream exon, leading to the reverse orientation of the included exons [17]. However, additional factors, such as IRAlus (inverted repeat Alu elements), within individual introns or across flanking sequences can lead to effective transcript circularization. Multiple circularized exons can be generated from a single gene locus where Alu elements are a significant factor [18]. Alu elements are widely distributed along intronic sequences, allowing multiple RNA pairings and competitive IRAlu formation and leading to alternative circularization [18,19].

### 2.2. Trans-Acting Elements

Many trans-acting factors are able to regulate circRNA generation under certain conditions. The presence of ADAR (adenosine deaminase acting on RNA)—theRNA-binding protein (RBP), which have the ability to deaminate adenosine to inosine in double-stranded RNA, leads to the destabilization of inverted repeats and hinders the circularization of the transcript [20]. Quaking (QKI) belongs to a group of RNA-binding proteins that enhance circRNA production during the epithelial–mesenchymal transition [21]. The mechanism of action includes binding to its target single-stranded RNA (ssRNA) motif in intron-flanking sequences, causing these ssRNAs to be circularized and dimerized, which brings two ends of the sequence into close proximity, thus resulting in circRNA formation [22,23]. Another circularization-regulating RBP is the FUS protein, a well-known splicing regulator that has also been shown to control the backsplicing processes of certain circular transcripts. FUS can act either as a canonical splicing activator or as a repressor, but it has also been reported that FUS can both enhance and repress backsplicing [24]. Furthermore, DHX9–ATP-dependent RNA helicase A has been shown to hinder circRNA production by unwinding the RNA structures formed by inverted Alu elements (IAEs) in the flanking regions of circRNAs. Moreover, DHX9 represses the expression of certain circRNAs by directly binding to Alu elements and regulating circRNA-producing genes, RNA processing, and translation [25]. Other interesting examples of proteins regulating circRNA generation are NF90 and its 110 isoform, NF110. Both RBPs promote circRNA production by stabilizing the intronic RNA pairs in the nucleus. On the other hand, upon viral infection, NF90/NF110 are rapidly exported to the cytoplasm, and the corresponding circRNA expression is decreased. Interestingly, the de-association of NF90/NF110 from circRNPs in the cytoplasm allows their binding to viral mRNAs to inhibit viral replication, suggesting the involvement of circRNAs in viral infection [26].

Circular transcript generation is connected over time and through machinery with canonical splicing, so this generation process has a strong correlation with the fate of pre-mRNA, as evidenced by the controlling mechanisms. Intronic sequences are major circularization-promoting factors responsible for the reduction in the linear splicing efficiency of flanking exons [27]. Moreover, the generated circRNAs can modulate parental gene expression, thereby affecting the amount of RNA and protein [11,28,29].

## 3. Stability of circRNAs

A distinctive feature of circRNAs from the other forms of RNA molecules is the presence of a covalent bond joining the 3′ and 5′ ends, forming a continuous loop which prevents degradation via RNA exonucleases. The covalent bond between the two ends of the sequence results in the lack of a 3′ poly-(A) tail and a 5′ cap structure [30]. The closed circle shape allows the circRNAs to be highly stable and abundant in the cytoplasm, exhibiting a longer half-life in comparison to their linear counterparts. The early studies of Harland and Misher in 1988 [31], followed by many others, demonstrated that circRNAs are extremely stable, with over 40-h half-lives. It was more recently proven that this half-life might range from 8 to 50 h, suggesting that additional properties and factors of circular transcripts regulate their stability [32]. Currently, very little is known about circRNA degradation mechanisms in vivo. In 2019, Liu et al. identified endonuclease L, which is known to globally degrade circRNAs upon activation [33]. Additionally, it was reported that m^6^A-containing RNAs, including circRNAs, might be degraded via the YTHDF2 (m^6^A reader protein), the HRSP12 (adaptor protein), and RNase P/MRP [34]. Some alternative degradation mechanisms are also known, such as the Ago2 slicer-dependent mechanism for CDR1as/ciRS-7 and the GW182 protein involved in circRNA degradation in many organisms [23].

## 4. Methodologies for Studying circRNAs

The structures of circRNAs are why most of their transcripts have remained elusive to researchers until recently. circRNAs are characterized by the presence of back-splice junctions (BSJs). Therefore, most of the techniques used for studying circRNAs are based on using head-to-tail junction detection to distinguish circular transcripts from their linear counterparts. Basic molecular biology techniques do not always allow the detection and correct estimation of the number and size of circular transcripts. circRNAs originate from various genomic locations and can differ in size even among their own isoforms, so it is difficult to separate them from other RNA species by electrophoretic mobility. Moreover, basic molecular techniques that require amplification and/or fragmentation have been shown to destroy the circular structures of circRNAs. The presence of covalent closed ends eliminates the value of techniques that rely on a polyadenylated free RNA end [35].

One of the most frequently used techniques that can confirm the presence of a circular transcript is the treatment of RNAse R, an exoribonuclease hydrolyzing RNA molecule from 3′ to 5′ end. The absence of the 3′ and 5′ ends renders circular transcripts resistant to enzymatic digestion, unlike linear transcripts. However, it has been shown that some circRNAs may also be susceptible to RNase R treatment [7]. Currently, increasingly more protocols based on the presence of BSJs have been developed, allowing the adaptation of basic molecular biology tools for the study of circRNAs [36]. Currently, it is possible to indicate the subcellular localization of circular transcripts using the fluorescent in situ hybridization method (FISH) by applying probes spanning the back-splice junction site [37]. Many studies also demonstrated the successful manipulation of circRNA expression levels using small interfering RNA (siRNA) and short hairpin RNA (shRNA) as a loss-of-function approach alongside circRNA expression vectors that overexpress circular transcripts [38]. 

Quantification methods using divergent primers allow the amplification of circular transcript sequences without the simultaneous amplification of the linear form [39]. RT-qPCR (reverse transcription quantitative polymerase chain reaction) is a technique used to validate the results of RNA sequencing data, which also has some technical challenges in the case of circRNA identification. As mentioned in the previous section, divergent primers spanning BSJ are used but can only amplify the circRNA sequence. Although RT-qPCR is a widely used technique, it can introduce errors when validating circular transcripts. Template switching during the RT step may result in the formation of by-product templates or the generation of long cDNAs consisting of sequence concatemers. Additionally, rolling circle reverse transcription (RT) may lead to the overestimation of circRNA in comparison with the RNA sequencing results [1]. Due to the given inconvenience, it is recommended to use the Northern blotting technique to confirm the existence of circRNAs, as Northern blotting does not rely on RT or PCR amplification [40].

Furthermore, the development of deep sequencing techniques allowed us to extend our knowledge of the circRNA field. The covalently closed structure of circRNAs has led to many problems in the detection of potential circular molecules during the analysis of high-throughput RNA sequencing results. The first algorithms developed to search for circRNAs were based on the algorithms used to study the phenomenon of alternative exon splicing. Using such tools, it was possible to detect a certain amount of circRNA in pre-existing data derived from total cellular RNA sequencing (RNAseq). However, such libraries often do not contain circRNAs whose existence was previously documented. This led to the development of new protocols for RNA-seq sample preparation, including exonuclease-based enrichment approaches, sequencing with longer reads, and the sequencing of ribosomal RNA (rRNA)-depleted RNA libraries [7,35].

## 5. Functions of circRNAs

circRNAs in eukaryotic cells were treated as splicing errors until the 2000s [41,42]. The dynamic development of research on the expression of circular transcripts has also raised questions about the functions of these puzzling transcripts [1]. According to the current state of knowledge, circRNAs may have various functions previously assigned to both non-coding and coding RNAs. In this section, we describe the functions of circRNAs both as regulatory factors for many elements of the transcriptome and as a template for protein production.

### 5.1. circRNA as Gene Expression Regulatory Factors

circRNA research has enabled scientists to identify circRNAs’ basic functions, especially their interactions with other molecules such as microRNA (miRNA) and RNA-binding proteins (RBPs). The deregulation of circRNA expression in diseases, especially cancer, is not without significance. The presence of numerous miRNA binding sites identified in the circRNA sequence demonstrated that circRNAs can act as miRNA binding molecules called miRNA sponges [43,44,45]. One of the best-known and earliest reported sponging circRNAs in humans is CDR1as, which has 70 conserved miR-7 binding sites. The binding of miRNAs to circRNAs prevents miRNAs from acting on their target molecules, thus leading to an increased expression of miR-7 target genes [46]. A similar effect occurs for RBPs, which can also serve as a target for sponging by circRNAs. A notable example of posttranscriptional regulation by the sequestration of RBPs is the MBL1 protein, which promotes the circularization of the second exon of its own pre-mRNA. circMbl biogenesis competes with mRNA production, resulting in the decreased expression of the MBL1 protein. A low level of the MBL1 protein leads to more efficient Mbl mRNA splicing and cannot promote circMbl generation, leading to a lower expression of circRNA. As the MBL1 expression increases, the protein binds to Mbl pre-mRNA to induce circularization. On the other hand, circMbl contains MBL protein binding sites that act as sponges and are able to sequester MBL proteins and hinder their production. circMbl is also able to control its individual expression via the sequestration of RBPs generated from its parental gene [47]. The natural occurrence of circRNAs acting as miRNA sponges led to the idea of creating artificial circular transcripts designed to reduce the levels of target miRNAs, which are known to impact pathological processes. The first example of synthetic circRNAs used to sponge miRNAs was provided by Liu et al. [48], indicating that miR-21 sponging can suppress the proliferation of gastric carcinoma cells.

It has been reported that certain exon-intron circRNAs, a class of circRNAs containing an intron retained between the exons, are able to regulate the expression of their parental genes via specific interactions with U1 snRNA (small nuclear RNA) [49]. Additionally, some circRNAs located in the nucleus bind with genomic DNA and form an RNA–DNA triplex involved in the regulation of DNA replication [50]. In addition to the functions mentioned above, circRNAs also have the potential to act as a template for protein production. 

Certain endogenous circRNAs can be translated to form protein or peptide isoforms in biologically significant amounts [51]. Two potential mechanisms of translation initiation have been suggested: the Internal Ribosome Entry Site (IRES) and the N^6^-methyladenosines (m^6^A)-mediated cap-independent mechanism [52,53].

### 5.2. circRNAs as a Template for Protein Synthesis

The characteristic features of circRNA structures, specifically the lack of the 3′ and 5′ untranslated regions (UTRs) that are replaced by a covalent bond joining the two ends, has led researchers to consider circRNAs as non-coding elements for a long period time. Recently, however, strong evidence for their protein-coding potential has emerged in the literature. Studies of their sequences indicate that a certain number of circRNAs contain the AUG initiation codon and “stop” codons followed by putative open reading frames (ORFs) [52,54,55].

#### 5.2.1. IRES-Mediated Translation

The first evidence for circRNAs’ coding potential was demonstrated in 1995 by Chen and Sarnow [56], who indicated that artificial circRNAs are able to initiate translation by recruiting the 40S ribosomal subunit and produce a biologically detectable amount of protein in vitro. However, the presence of Internal Ribosome Entry Sites (IRESs) or m^6^A modifications has been a necessary element for translation initiation in some groups of circRNAs (Figure 1). As a result of artificial circRNA translation, the long repeating polypeptide chain was obtained from the continuous open reading frame without a stop codon [56]. It was also indicated that IRESs are one of the necessary elements for cap-independent translation initiation, which is one of the naturally occurring mechanisms of alternative mRNA translation initiation during the physiological stimulation of cell differentiation, synapse network formation, and stress conditions [57]. IRESs contain RNA elements that are typically found in the 5′ UTR of mRNA because 5′ cap recognition is necessary for the initiation complex assembly [58]. In the cap-independent mechanism of translation initiation, a non-canonical eIF4G protein needs to recognize IRESs to initiate the eIF4 complex assembly in order to initiate the translation [59]. However, the locations of these elements are not limited, which is highlighted by the presence of IRESs within the circRNA sequence, especially considering the number and variability of circRNAs and their isoforms [60]. IRES sequences form secondary sequences on the RNA that initiate translation even without canonical translation initiation factors present; these factors are replaced by IRES trans-acting factors [57].

Interestingly, circRNA sequences can be investigated not only to find potential miRNA and RBP binding sites but also to obtain information on the presence of potential IRES sequences, which are suspected to be translated in vivo. The CircInteractome web tool revealed the presence of IRESs in several circRNA sequences, reporting that functional proteins might be generated therein. These IRES sequences might also serve as putative binding sites for several RBSs, including human antigen R (HuR) and polypyrimidine tract binding (PTB) protein, which have been reported to modulate IRES-driven translation [61]. The role of IRES in translation initiation was also indicated in experiments engaging artificial circRNAs. The generation of artificial circRNA engineered as a minigene containing a single exon was also reported [62]. This minigene encoded two GFP (green fluorescence protein) fragments in reverse order. An IRES located upstream of the start codon served as a translation initiation element. Thus, it has been shown that an engendered sequence can be backspliced with great efficiency, which was an obstacle in previous experiments. Additionally, it was found that canonical splicing factors can also regulate backsplicing but with different principles from canonical splicing [62]. circRNA engineering has created a system for inducible circRNA formation. To obtain the proper result, a 3′ intron of a circRNA expression cassette was designed to contain a hairpin targeted of endoribonuclease Csy4. The inducible mechanism relies on the presence of Csy4; in its absence, canonical splicing is favored, leading to mRNA translation. The presence of the Csy4 enzyme leads to pre-mRNA cleavage in the hairpin position and removes a competitive splice site, resulting in the circularization of the transcript.

#### 5.2.2. m^6^A-Mediated Translation

N^6^-methyladenosine (m^6^A) is considered the most common internal modification of mRNAs. m^6^A RNA methylation regulates basic cellular events, such as tissue development, DNA damage response, and sex determination, but also plays a role in tumorigenesis [63,64,65,66]. Methylated adenosine residues in the form of N^6^-methyladenosines (m^6^A) present in the 5′ UTR also serve as another cap-independent translation mechanism of circRNAs. The N^6^-methylation of adenosine impacts mRNA and circRNA translation under heat shock stress [66,67]. The translation of m^6^A-containing circRNAs requires YTHDF3, an m^6^A reader protein that can directly interact with eIF4G2. eIF4G2 recognizes the IRES and initiates the eIF4 complex assembly, which leads to the initiation of translation [59]. Surprisingly, it was indicated that a specific pattern of m^6^A modification in circRNAs exists. This pattern is also cell-type specific and distinct from mRNAs. In 2017, Zhou et al. [68] identified the circRNAs specific to human embryonic stem cells (hESC) and HeLa cells, revealing common and cell-type-specific expression patterns. Interestingly, m^6^A circRNAs show similar expression levels when expressed in both cell lines. The differences in the lengths of m^6^A circRNAs and non-m^6^A circRNAs highlight that the lengths of all exons in m^6^A circRNAs are longer than those in circRNAs without m^6^A modifications [68].

#### 5.2.3. Rolling Circle Amplification-Mediated Translation

To date, many circRNAs have been associated with polysomes, leading to the conclusion that a significant number of these molecules can be translated [59,69,70]. Both IRES- and m^6^A-mediated translation are considered significant and common elements of circRNA translation initiation. Rolling circle amplification (RCA) has been proposed as another putative mechanism of circRNA translation [71,72]. Certain circRNAs contain infinite open reading frames that allow translation in a manner similar to rolling circle amplification. In 2015, Abe et al. [71] designed a circRNA sequence with an infinite open reading frame to test its translation potency in the eukaryotic system. Translation was conducted in rabbit reticulocyte lysate and HeLa cells without any of the aforementioned elements necessary for translation initiation. Instead, the authors used the Kozak consensus sequence that contained an initiation codon, a key player of translation initiation in eukaryotic systems. The results showed that circRNAs with infinite ORFs can be translated via RCA; in this case, the presence of an IRES sequence or N^6^-methyladenosine modification is not necessary for protein synthesis.

Despite the comprehensive knowledge and experience in the field of circRNAs, more sophisticated experimental methods that facilitate the efficient identification of translatable circRNAs and their products are still required.

## 6. Relevance of circRNAs for Human Diseases

The features of circRNAs, such as their stability and tissue-related abundance, make them promising potential biomarkers for human diseases. Apart from their presence in tissues of different origins, circRNAs are also enriched in bodily fluids, including blood, saliva, cerebrospinal fluid, and urine. circRNAs can be specifically detected in the free-floating cells inside these bodily fluids, such as circulating blood cells and circulating tumor cells. Moreover, they are present in the extracellular vesicles circulating in blood and other bodily fluids [73].

### 6.1. circRNAs in Cancers

Research into the role of circRNAs in disease processes covers almost all fields of medicine. The dysregulation of circRNAs is most widely described in oncology, emphasizing their potential roles as promising prognostic, diagnostic, and therapeutic molecules. Several circRNAs, such as hsa_circ_0000190 and hsa_circ_002059 downregulation, correlate with the gastric cancer tumor grade and the presence of distal metastasis [74,75]. Moreover, circRNAs may serve as useful diagnostic tools for hepatocellular carcinoma. circZKSCAN1 and circ-ITCH have different expressions compared to healthy hepatic tissue [76,77]. Most importantly, changes in the expression profiles of circRNAs are associated with tumor invasiveness and overall survival and can be detected in both the tumor tissue and plasma samples collected from patients [8,73]. In lung carcinoma, early detection is crucial for effective treatment. Hsa_circ_0013958 upregulation is observed in both tissue samples and plasma collected from patients and seems to be a perfect candidate for non-invasive screening [78]. Similarly, in other types of malignancies, such as breast cancer and colorectal cancer, circRNAs are very likely to become specific biomarkers and can be used in novel therapeutic strategies. Vo et al. [79] examined over 800 human cancer samples and developed a compendium, MiOncoCirc, which is an open database of detected circRNAs. Their analysis revealed the correlation between dysregulated host genes and derived circRNAs. The authors also divided dysregulated circRNAs into groups based on tissue specificity. Ng et al. [80] provided a thorough analysis of literature covering the circRNA-miRNA-mRNA regulatory network in cancer development. For example, circFOXO3 acts as a sponge for multiple miRNAs in breast cancer cells (miR-22, miR-136, miR-138, miR-149, miR-433, miR-762, miR-3614-5p, and miR-3622-5p) and inhibits tumor growth and angiogenesis.

### 6.2. circRNAs in Other Diseases

circRNA’s well-explored activity as an miRNA sponge impacts major cellular processes not only in carcinogenesis but also in cardiovascular [81], neurological [82], ophthalmological [83], and autoimmune diseases [84].

In cardiovascular disorders, heart-related circRNA (HRCR) appears to play an important role in cardiac hypertrophy via miR223-5p sponging. In a previous study, virally mediated HRCR overexpression resulted in miR-223-5p downregulation and, therefore, the protection of the Apoptosis Repressor (ARC) activity and the modulation of cell proliferation [85]. Circulating hsa_circ_0005870 is upregulated in plasma samples from hypertensive patients [86]. Similarly, hsa_circ_0124644 is upregulated in patients with coronary arterial disease [87], and circSLC8A1-1 overexpression correlates with heart failure [85].

Apart from tissue specificity, circ-Foxo3 interacts with miRNAs in cancer cells and binds with proteins in cardiac tissue [81]. Similar to circ-Foxo3, circ-ZNF609 shows different activities in different tissues. In myoblasts, as mentioned before, circ-ZNF609 has the ability to produce the short protein. In retinal tissue, it sponges miR-615, thereby affecting retinal gliosis and retinal ganglion cell survival in glaucoma [83]. These activities demonstrate the wide potential of these particles that is not yet fully explored. Recent data also showed the involvement of circRNAs in eclampsia, diabetes, rheumatoid arthritis, and tuberculosis [88,89,90,91], highlighting the remarkable potential of circRNAs as molecules of therapeutic importance.

The idea of using circRNAs as biomarkers is certainly encouraging, but there is still much to be explored. As some circRNAs change their expression profiles only in tissues but not in plasma, non-invasive sample collection may not be an option. In this case, circRNA profiling from tumor tissues collected from diagnostic biopsies may yield additional information about the prognosis and best course of treatment.

## 7. Proteins Derived from circRNAs—Course of Action in Human Pathologies

The relevance of circRNAs in human pathologies has already been greatly acknowledged, as shown in the previous section. However, one of the other functions of circRNAs is their protein-coding potential, which raises the question about the functions of their products. This issue remains poorly recognized and needs to be more thoroughly investigated. Here, we summarize current studies on the peptides and small proteins encoded by circRNAs and their potential courses of action (Table 1).

Bagchi [101] performed an analysis of 21 circRNAs with documented coding potential. He used their amino acid sequences to create three-dimensional structures and revealed that circRNA translation products create domains that are identical to previously reported proteins, some of which can be associated with different diseases. For instance, two proteins derived from hsa_circ_02276 and hsa_circ_04264 are involved in the onset of oligodendroma. Both may contribute to cytoskeletal formation and organelle organization and present spectrin and dynactin domains. The protein that originates from hsa_circ_12152 has a nearly 92% sequence identity with Tropomyosin receptor kinase A (4AT5) and is involved in glioblastoma development. The results of the analysis of the structural similarities between circRNA encoded peptides and well-known particles suggest that the main activity of these peptides is linked to linear-mRNA derived analogues [101].

### 7.1. circRNAs-Derived Proteins as a Decoy

Pathogenesis is caused by disturbances at many levels in the network of cellular pathways. Proteins derived from circRNAs may act as rescuers for their counterparts translated from linear RNAs, thereby protecting crucial cell-cycle regulators from degradation. The full-length SNF2 histone linker PHD RING helicase (SHPRH) regulates genome stability by targeting proliferating cell nuclear antigen (PCNA). Mutations in the SHPRH gene occur in various cancer cell lines, including those of ovarian cancer and melanoma [102,103]. circ-SHPRH forms by backsplicing exons 26–29 of the *SHPRH* gene and comprises 440 nucleotides with a tandem of STOP codons—UGAUGA—in the junction site. Using the overlapping genetic code, circ-SHPRH is entirely translated, creating a 146-aa protein [92]. The downregulation of SHPRH-146aa in glioma cells results in intensified carcinogenesis and a decreased expression of the full-length SHPRH protein. However, the up-regulation of SHPRH-146aa prolongs the half-life of full-length SHPRH, suggesting that SHPRH-146aa can act as a decoy protein, protecting SHPRH from degradation because both SHPRH-146aa and SHPRH share the same amino acid fragment at the C-terminus. The biological effect of SHPRH-146aa dysregulation was observed in tissues collected from glioblastoma patients. In 81% of the samples, SHPRH-146aa was down-regulated, and its lower levels correlated with a shorter survival time. The over-expression of SHPRH-146aa in the U251 and U373 cell lines reduces cell proliferation, but not in cells with full-length SHPRH knockdown. This supports the hypothesis that the SHPRH-146aa mechanism of action depends on full-length SHPRH [92]. The down-regulation of circ-SHPRH expression results in the increased proliferation, migration, and invasion of cancer cells [104,105,106].

**Figure 2:**
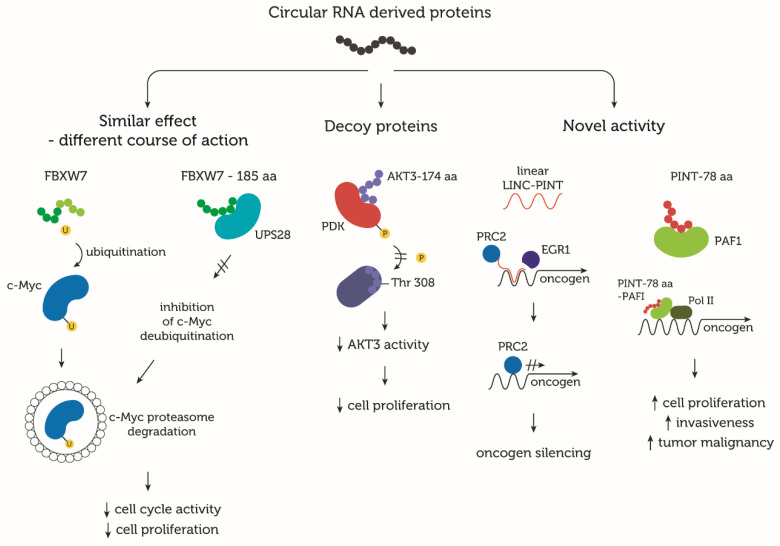
Examples of circRNA-derived proteins and their activity. Some proteins originating from circRNAs share the same activity as their linear-derived counterparts, but their goals are achieved in different manners. FBXW7-185aa arrests cell cycle and cell proliferation, but, in contrast to FBXW7, it cannot directly interact with c-Myc. It affects c-Myc ubiquitination via the activation of the deubiquitinating enzyme, UPS28. Tided UPS28 loses its activity, thus enhancing the c-Myc ubiquitin-dependent proteasome degradation [96]. AKT3-174aa binds to phosphorylase phosphoinositide-dependent kinase (PDK) and acts as a decoy protein for AKT3, preventing it from undergoing phosphorylation and activation [93]. However, some proteins may act independently of their linear analogues. PINT-78aa anchors PAF1, recruits Pol II to the oncogene promotor site, and activates transcription, resulting in enhanced cell proliferation and invasiveness. At the same time, a linear form of the long intergenic non-protein coding RNA, p53 induced transcript (LINC-PINT) silences oncogene expression by interacting with PRC2 and EGR1 proteins (PRC2-polycomb repressive complex 2) [113].

The RTK/PI3K/AKT pathway is also under investigation due to its significant role in carcinogenesis. An enhanced activation of protein kinase B (AKT) occurs in over 70% of glioblastoma patients and results in tumor cell proliferation and malignant transformation through multiple targets [107]. Three described isoforms of AKT share similar sequences but have isoform-specific functions. The AKT3 isoform strongly correlates with neuronal development and astrocyte differentiation but is also dysregulated in various cancers [108,109]. To date, the function of several circRNAs derived from the *AKT3* gene have been explored [110,111]. Huang et al. [112] identified a circRNA originating from exons 8–11 of the *AKT3* gene that is overexpressed in cisplatin-resistant gastric cancer. The authors reported that circAKT3 (hsa_circ_0000199) promotes the over-activation of the phosphatidylinositol 3′-kinase(PI3K)-Akt signaling (PI3K-Akt) pathway by sponging miR-198. Interestingly, Xia et al. [93] did not observe this particular circRNA in neuronal tissue, highlighting the organ-specific circRNA expression patterns. circAKT3 (hsa_circ_0017250), described by Xia et al., arises from the circularization of exons 3–7; it is 524 nt long and contains an ORF with overlapping start-stop codons—UAAUAG. The encoded protein AKT3-174aa acts as a tumor suppressor and is down-regulated in highly malignant glioblastoma cells. Similar to SHPRH-146aa, AKT3-174aa is a decoy protein. By interacting with phosphoinositide-dependent kinase (PDK), AKT3-174aa inhibits the phosphorylation of AKT at the Thr308 position, thereby reducing its activity (Figure 2). This, in turn, inhibits cell proliferation and invasiveness in glioblastoma cells in vitro and in vivo [93].

Another protein that is dysregulated in cancer is β-catenin-370aa. Its full-length counterpart, β-catenin, is actively involved in liver cancer development via the regulation of the Wnt pathway; the copy number of its gene locus is amplified in liver cancer tissue [114]. circRNA originating from the β-catenin gene contains six exons and is 1129 nt long. This circRNA comprises an ORF, and its START codon is shared with linear β-catenin mRNA, but its STOP codon forms after circularization. circβ-catenin is more stable in the cytoplasm than its linear cognate and serves as a template for translation. β-catenin-370aa shares a similar function in carcinogenesis but with an alternative mode of action. circβ-catenin is highly expressed in liver cancer cells compared to healthy tissue, and its knockdown results in a reduction in cancer cell growth and migration. The suppressive effect on carcinogenesis is achieved by β-catenin-370aa rather than the circRNA itself. Acting as a decoy protein, β-catenin-370aa binds at the N-terminus with GSK3β, a ubiquitin ligase, and protects the full-length β-catenin from proteasome-dependent degradation. This, in turn, enhances the Wnt/βcatenin pathway’s activity. Silencing circβ-catenin reduces the β-catenin expression, thereby inhibiting cell invasiveness, which promotes circRNA as a favorable candidate for therapeutic targets [53,94].

### 7.2. Similar Effect, Different Course of Action

A different function of circRNA-derived protein was described by Yang et al. [96]. For FBXW7-185aa, small proteins can achieve the same effect as their linear-derived analogues but in a different manner. An F-box with seven tandem WD40 proteins (FBXW7) suppresses multiple oncogenes, such as c-Myc, via ubiquitination and the ubiquitin–proteasome system (UPS) degradation (Figure 2). Disturbances in FBXW7 expression lead to uncontrolled cell growth and have been described in numerous human malignancies [115,116,117]. FBXW7-185aa has similar tumor-suppressive abilities to its full-length counterpart but in a different manner. FBXW7-185aa originates from circFBXW7, which is a 620 nt product of backsplicing exons 2 and 3 of the *FBXW7* gene and lacks the C-terminus WD40 domain. FBXW7-185aa cannot directly bind to c-Myc, causing its ubiquitination, but FBXW7-185aa achieves this effect via the inhibition of the de-ubiquitinating enzyme, USP28. The expression of FBXW7-185aa and circFBXW7 decreased in glioblastoma samples, correlating with tumor malignancy and overall patient survival time. The FBXW7-185aa up-regulation in glioblastoma cell lines reduced the cell-cycle activity and cell proliferation. However, the FBXW7-185aa knockdown increased invasiveness and hastened growth [96].

Ye et al. [95] conducted similar observations on triple-negative breast cancer. The overexpression of FBXW7-185aa resulted in c-Myc degradation and reduced the proliferation and migration abilities of breast cancer cells. Additionally, the authors reported that circFBXW7 inhibits tumor growth by sponging miR-197-3p, highlighting the multifunctional potential of circRNAs.

Proteins derived from circRNAs may also interact directly with their full-length versions, thereby enhancing their activity. The circLgr4-peptide encoded by circLgr4 facilitates the Wnt pathway by binding with LGR4. Leucine-rich repeat-containing G-protein coupled receptor 4 (LGR4) is involved in multiple cellular processes, including cell self-renewal and invasion. It activates Wnt/beta-catenin signaling by binding to the Wnt agonist, R-spondins (RSPO1), and stabilizing the Frizzled receptors (FZD7 and FZD10) [114,118]. The over-expression of circLgr4 correlates with colorectal stem cell invasiveness and malignancy, but only in an LGR4-dependant manner [97].

### 7.3. Novel Independent Activity

Circularization allows the creation of novel alternative versions of proteins that differ in action from their full-length counterparts. Zhang et al. [98] identified an 87-aa long peptide originated from the circular form of exon 2 of the long intergenic non-protein-coding RNA p53-induced transcript (LINC-PINT). PINT87aa acts independently of its circRNA and the linear form of LINC-PINT. Located mostly in the nucleus, PINT87aa regulates the transcriptional elongation of multiple oncogenes by interfering with the polymerase-associated factor 1 (PAF1), which recruits RNA polymerase II (Pol II). The up-regulation of PINT87aa expression does not affect the PAF1 levels themselves but enhances the interactions between PAF1 and its target gene promoters, *c*-*Myc* or *CPEB1*, thereby regulating cell-cycle acceleration. PINT87aa downregulation results in the loss of the proper localization of PAF1, suggesting that PINT87aa may be the anchor for a PAF complex on the target promoter, thus reducing the Pol II-dependent mRNA elongation (Figure 2) [98]. The down-regulation of PINT87aa has been associated with high tumor invasiveness; grade IV glioblastoma showed the lowest expression of this protein compared to healthy tissue. Reduced levels of PINT87aa and its circRNA were also observed in other malignancies, such as breast, hepatic, and gastric carcinomas, in contrast to regular cell lines [119]. PINT87aa is a potential candidate for carcinogenesis biomarkers and novel therapeutic strategies [53].

PPP1R12A-73aa also presents a different expression profile and function from the full-length protein encoded by linear mRNA. In contrast to PPP1R12A, PPP1R12A-73aa exerts pro-carcinogenic activity, and the expression profiles of circRNAs in colon cancer cells revealed an increased level of circPPP1R12A (hsa_circ_0000423) compared to healthy tissue. circPPP1R12A contains 216 nt ORFs. The small protein PPP1R12A-73aa is transcribed by using overlapping initiation and termination codons created after circularization. Overexpression was strongly correlated with tumor malignancy and shortened overall survival. The protein’s influence on colon cancer cells is correlated with PPP1R12A-73aa up-regulation but not with the over-expression of its circRNA. This protein interacts with the Hippo-YAP signaling pathway, resulting in enhanced cell proliferation and invasiveness because the YAP protein is one of the transcriptional regulators involved in tissue growth, regeneration, tumor progression, and metastasis [99].

### 7.4. Unknown Function

The overexpression of circZNF609 affects myoblast proliferation, causing a delay in muscle differentiation. circZNF609 originates from the circularization of the second exon of ZNF609 pre-mRNA. The open reading frame of 753 nucleotides starts with the putative AUG of the host gene and exceeds the splice junction by three nucleotides. Legnini et al. [54] investigated the coding potential of circZNF609. The authors observed that the flagged protein derived from circZNF609 lacks the zinc-finger domains and demonstrates mainly nuclear localization in contrast to its analogue derived from linear mRNA. Translation activation was elicited by a heat shock, suggesting that cap-independent translation might be regulated by cellular stress in this case. Given the differences, it is still unclear whether the protein derived from circZNF609 carries similar properties to its counterpart linear RNA [54,120].

The miRNA sponge role of circZNF609 has been well documented in different disorders, including renal, nasopharyngeal, and gastric cancers. Changes in the ZNF609 circRNA expression correlate with various cellular processes, but it is unknown whether the main modulating potential is due to the miRNA sponging, circRNA-encoded proteins, or another possible mechanism of action [121,122,123,124].

### 7.5. Other

Apart from circRNAs originating in host genes, viral circRNAs are also involved in carcinogenesis. Human papillomavirus produces circE7, a 472-nt oncogene comprising the ORF for the E7 protein. circE7 is localized mostly in the cytoplasm and can be modified by N^6^-methyloadenosine (m^6^A). In cervical cancer cells, the reduced E7 protein levels caused by circE7 knockdown result in the inhibition of cancer cell growth both in vitro and in tumor xenografts [53,100,125]. On the other hand, in anal squamous cell carcinoma and HPV-positive head and neck carcinomas, the elevated levels of the circE7 and, therefore, E7 proteins were linked to improved overall survival. This inverse correlation may be caused by an enhanced immunological response to viral protein overexpression [126]. Interaction with miRNAs is an additional mechanism of action of HPV-originated oncoproteins. E7 upregulates miR-20a expression, which in turn results in reduced cell proliferation and invasion in oral squamous cell carcinoma [127]. The potential role of the circE7 or E7 proteins as positive prognosis biomarkers has yet to be established.

## 8. circRNA-Protein Interaction

**Figure 3:**
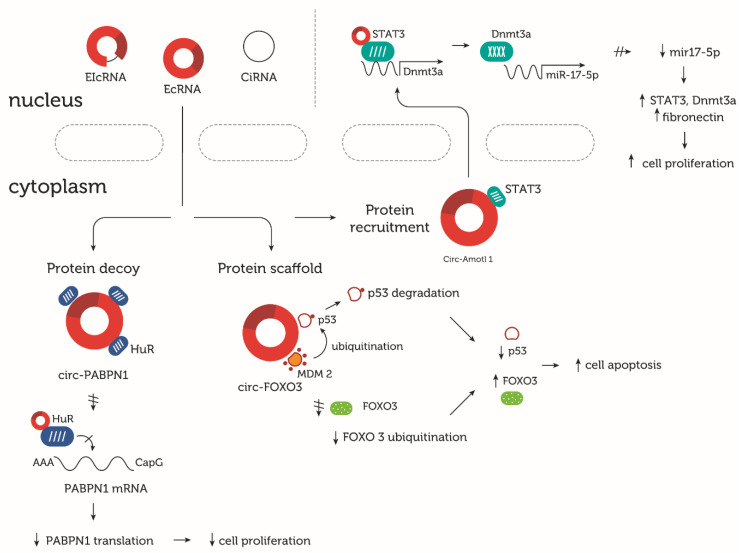
circRNAs acting as decoy, scaffold, and recruiter molecules. Depending on the conditions, some circRNAs may exert several activities. circ-PABPN1 can decoy the human antigen R (HuR) protein in HeLa cells, thereby preventing HuR from attaching to PABPN1 mRNA, inhibiting PABN1 mRNA translation and resulting in decreased cell proliferation [134]. The circ-FOXO3 scaffold p53 and MDM2 cause ubiquitin-dependent p53 degradation and, at the same time, decrease FOXO3 ubiquitination. Reduced p53 and increased FOXO3 levels result in enhanced cell apoptosis in cancer cell lines [132]. circ-Amolt1 recruits STAT3 and changes its location from the cytoplasm back to the nucleus in skin fibroblasts. The circ-Amotl1-STAT3 complex promotes Dnmt3a transcription, which in turn inhibits the miR17-5p production. Decreased miR17-5p results in STAT3, Dnmt3a, and fibronectin overexpression, thus activating cell proliferation, which is critical in the wound healing process [130].

The multifunctionality of circRNAs extends beyond their miRNA sponging and protein-coding abilities. One of the main oncogenes, c-Myc, which is regulated by FBXW7-185aa, interacts with circ-Amotl1 in breast cancer cells. An analysis of the potential binding sites and secondary structure of circ-Amotl1 uncovered the possible docking site for this circRNA in the central region of c-Myc. The overexpression of circ-Amotl1 results in cancer progression and metastasis through c-Myc stabilization and retention in the nucleus, with circ-Amotl1 as the decoy molecule (Figure 3) [128]. circ-Amotl1 may also act as a scaffold particle, interacting with PDK1 and AKT1 and facilitating their nuclear translocation and proper localization. In vivo, circ-Amotl1 reduces apoptosis and promotes AKT cardiomyocyte survival, exhibiting protective activities against Doxorubicin (Dox)-induced cardiomyopathy [129]. Another function of circ-Amotl1 is protein recruitment to precise cellular locations (Figure 3). circ-Amotl1 recruits Signal Transducer and Activator of Transcription 3 (STAT3) from the cytoplasm to the nucleus, which stabilizes the binding of STAT3 to the DNA (cytosine-5-)-methyltransferase-3-alfa (Dnmt3a) promoter. This leads to increased cell adhesion, migration, and proliferation, playing an essential role in wound healing (Figure 3) [130]. A change in the protein’s location from the cytoplasm to the nucleus also occurs under cooperation between circ-FECR1 and Ten-Eleven Translocation methylcytosine dioxygenase 1 (TET1) in breast cancer cells [131].

circ-Foxo3 acts as a scaffold binding with two proteins, p53 and MDM2, thereby modulating MDM2 ubiquitination, which, in turn, enhances MDM2-dependant p53 ubiquitination (Figure 3). Altogether, this results in prompt p53 degradation. circ-Foxo3 also protects Foxo3 from ubiquitination and deterioration in an MDM2-dependant manner, thus promoting apoptosis in cancer cell lines [132]. circ-Foxo3 and its protein-binding capacity also regulate senescence processes in cardiac tissue. circ-Foxo3 promotes cellular ageing by arresting the senescence-related proteins ID1 and E2F1 and the stress-related proteins HIF1a and FAK in the cytoplasm. The decreased levels of these proteins in the nucleus result in enhanced programmed cell death. The knockdown of circ-Foxo3 is a potential therapeutic approach for myocardial protection [133].

It has also been suggested that circPABPN1 (hsa_circ_0031288) serves as a decoy for HuR protein and inhibits polyadenylate-binding nuclear protein 1 (PABPN1) translation in HeLa cells (Figure 3) [134].

## 9. Conclusions and Future Perspectives

In the light of new reports, the role of circRNAs is becoming increasingly clear, but many issues and connections are still a mystery. Although circRNAs are still mainly considered as miRNAs or RBP sponges, here we revealed some unusual and less-understood functions of circular transcripts, primarily emphasizing their emerging coding potential. Over the past few years, many techniques have been developed to solve the puzzle of circRNAs encoding proteins. Currently, circRNAs’ functions in pathologies are being extensively studied, most notably their intensive identification using deep sequencing methods. Despite the significant advances in high-throughput deep sequencing technologies, there is still no gold standard for data analysis in the circRNA field [1,50]. It is advisable to use more than one tool for analysis, as various tools work with different efficiencies to balance false positives and false negatives. Moreover, some circRNAs require gene annotation while others work de novo, which may lead to difficulties in identifying all circular transcripts [135,136]. 

The most frequently raised issues are the properties of the circular transcript that allow the formation of proteins, a decisive factor for certain circRNAs to have coding potential. In contrast, other circular transcripts do not serve as a template for protein synthesis. Studying these issues yielded a new approach to manipulate circRNA levels by creating synthetic circRNAs, which have been shown to have therapeutic value in animal disease models, thereby presenting an alternative to DNA expression systems as a mode of gene therapy delivery. Several reports have illustrated the significant role of circRNAs in cancer and non-cancerous diseases. The circRNA expression patterns in cancer tend to be downregulated in many cases, which may potentially offer a field for the introduction of synthetic circRNA technology. Circular molecules may also be able to bind to more proteins, enabling them to act as protein sponges, decoys, stabilizers, scaffolds, protein recruiters, and protein products themselves. Circular scaffolds like circRNAs may be crucial for proteins to assemble in a specific spatial orientation. circRNAs can specifically influence the protein of interest, impact the protein abundance in various cellular compartments, and recruit proteins to a precise location. An imbalance in a protein’s presence or activity can change cell behavior, leading to pathologic conditions. The ability of circRNAs to interact with various targets suggests that they might be involved in the onset and progression of pathological processes. In the future, this revolutionary approach may serve as a tool to control oncomiRs in tumorigenesis and tumor progression. The production of artificial circRNAs allows the modulation of another function of circular transcripts: protein-coding. Circular transcripts may constitute a new class of diagnostic biomarkers and could be potential targets for modern treatment. Although much remains to be discovered, the current knowledge and emerging technologies suggest that we are entering a new era in the therapies of many diseases.

## Figures and Tables

**Table 1:** Summary of proteins encoded by circRNAs in human diseases.

Function	Protein	circRNA	Expression in Diseases	Cellular Pathways	References
Decoy proteins	SHPRH-146aa	circSHPRH (hsa_circ_0001649)	Downregulation in glioblastoma	Proliferation, migration, invasiveness	[92]
	AKT3-174aa	circAKT3 (hsa_circ_0017250)	Downregulation in glioblastoma	Proliferation, invasiveness	[93]
	β-catenin-370aa	circβ-catenin (hsa_circ_0004194)	Up-regulation in liver cancer	Invasiveness	[94]
Similar activity or different courses of action	FBXW7-185aa	circFBXW7	Downregulation in glioblastoma Downregulation in breast cancer	Proliferation, cell migration	[95,96]
	circLgr4-pept	circLgr4 (hsa_circ_02276)	Upregulation in colorectal cancer	Cell self-renewal, invasiveness	[97]
Novel activity	PINT87aa	circLINC-PINT (hsa_circ_0082389)	Downregulation in glioblastoma	Invasiveness	[98]
	PPP1R12A-73aa	circPPP1R12A (hsa_circ_0000423)	Upregulation in colon cancer	Cell growth, regeneration	[99]
Unknown	ZNF609-derived protein	circZNF609 (hsa_circ_0000615)	Overexpression in myoblasts Duchenne disease	Cell maturation	[54]
Other	E7	circE7	Upregulation in cervical cancer, Anal squamous cell carcinoma, HPV-positive head and neck carcinoma	Cell growth	[100]
